# Orthodontic treatment of severe anterior open bite and alveolar bone defect complicated by an ankylosed maxillary central incisor: a case report

**DOI:** 10.1186/1746-160X-10-47

**Published:** 2014-11-21

**Authors:** Feiou Lin, Hao Sun, Linjie Yao, Qiushuo Chen, Zhenyu Ni

**Affiliations:** Orthodontic Department, School of Stomatology, Wenzhou Medical University, Wenzhou, China; Pedodontic Department, School of Stomatology, Wenzhou Medical University, Wenzhou, China; Orthodontic Department, School of Stomatology, Wenzhou Medical University, No. 113 West Xueyuan Road, Wenzhou, Zhejiang China

**Keywords:** Orthodontic, Open bite, Alveolar bone defect, Dental trauma

## Abstract

Incisor trauma is common in children, and can cause severe complications during adolescent growth and development. This report describes the treatment of a 16-year-old patient with severe anterior open bite due to ankylosis of the maxillary left incisor after dental trauma as an 8-year-old. No examination or active treatment was undertaken until he was 16 years old. Clinical examination revealed that the maxillary left incisor was severely intruded accompanied by a vertical alveolar bone defect. Orthodontic treatment combined with surgical luxation took 3 years and 7 months. During treatment, the intruded incisor was moved to the occlusal level and the alveolar bone defect was restored, achieving normal occlusion. After two years of retention, the maxillary left incisor was retained in a stable normal position with a slightly reduced overbite. This case demonstrates that surgical luxation with orthodontic traction can be an effective approach, especially when the ankylosed tooth has a single root. Long-term monitoring of orthodontic stability and the maintenance of periodontal health are crucial in the post-treatment period.

## Background

The majority of dental injuries occur in children, and luxation of the permanent teeth is the most frequent dental injury in children aged 6 to 12 years [1]. Ankylosis is a common complication after traumatic events such as dental luxation, and may lead to local destruction of the periodontal ligament. External replacement resorption (ankylosis-related) is the result of injury to the innermost layer of the periodontal ligament and possibly the cementum. The healing process takes place from the adjacent alveolar bone, causing ankylosis [2–4]. Dentoalveolar ankylosis is an eruption anomaly defined as the union of the tooth root to the alveolar bone, with local elimination of the periodontal ligament [5]. An ankylosed tooth can lead to serious clinical problems such as vertical alveolar bone loss, tipping of adjacent teeth, midline deviation, impaction of the ankylosed tooth and supra-eruption of the opposing tooth [6, 7]. Clinical diagnosis of ankylosis is based on typical metallic sounds upon percussion, lack of tooth mobility and dental infra-occlusion. The most reliable sign is infra-occlusion, because only one third of reported patients exhibit a metallic sound, and only one third of radiographs show loss of the periodontal ligament space [8, 9]. Mullally [10] suggested that lack of orthodontic movement can confirm the diagnosis of ankylosis. The etiology of dental ankylosis includes: (1) trauma; (2) genetic factors; (3) local metabolic anomalies; (4) deficiency of alveolar bone growth; and (5) abnormal pressure of the soft tissues [11]. Anterior open bite malocclusion develops as a result of the interplay of many different etiologic factors [12], and is usually difficult to treat orthodontically. Treatment methods include orthognathic surgery, the multiloop edgewise arch wire technique, microscrew implant anchorage, and anterior vertical elastics [13–16]. Several surgical treatment protocols are designed to extrude an ankylosed tooth, such as single tooth osteotomy, surgical luxation, and distraction osteogenesis [17–19].The purpose of this case report is to illustrate the treatment of a severe anterior open bite and alveolar bone defect complicated by an ankylosed maxillary central incisor.

## Case presentation

**Figure 1:**
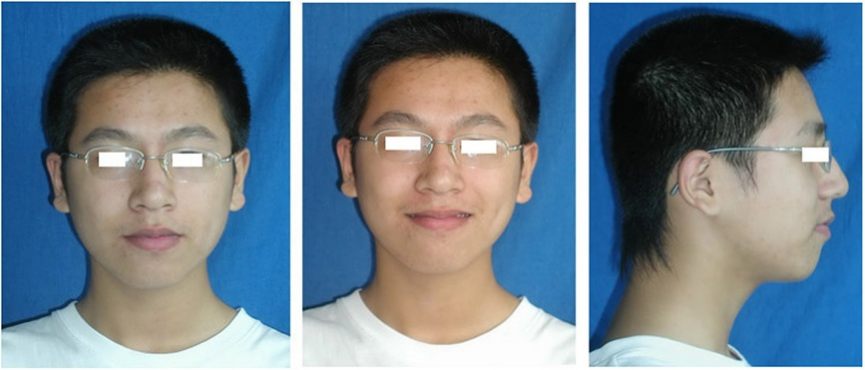
**Pre-treatment facial photographs showing how the patient was reluctant to smile because he was ashamed of the appearance of his anterior teeth.**

**Figure 2:**
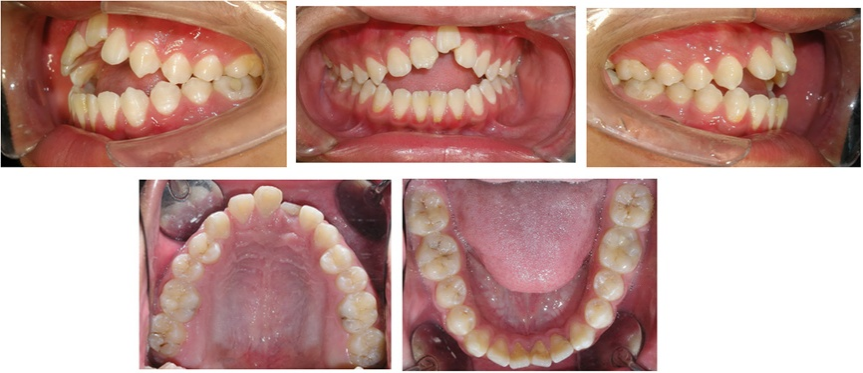
**Pre-treatment intraoral photographs showing a severe anterior open bite from the left maxillary canine to the right lateral incisor.** The maxillary left central incisor was severely infra-occluded and the right central incisor had a fractured crown.

A 16-year-old boy presented with anterior open bite and infra-occlusion of the maxillary left incisor. His anterior teeth had been injured in a fall when he was 8 years old. He had no dental treatment before attending the orthodontic department. According to the patient, his open bite had developed gradually. His facial profile was straight with a slightly retruded mental region. Facial analysis showed symmetry and a good balance between the facial thirds. The patient did not like to smile as he was ashamed of his teeth (Figure 1). He also had a compensatory tongue thrust habit caused by the anterior open bite.An intraoral examination (Figure 2) showed that the patient had a severe anterior open bite extending from the left maxillary canine to the right lateral incisor. The molar relationship was Class I, and there was a small space between the maxillary right lateral incisor and the canine. The maxillary midline had shifted to the left. The maxillary left central incisor was severely infra-occluded and the adjacent teeth were inclined. The crown of the maxillary right central incisor had been fractured, and the endodontist found that the pulp of the right central incisor was necrosed, although the other incisors were vital.

**Figure 3:**
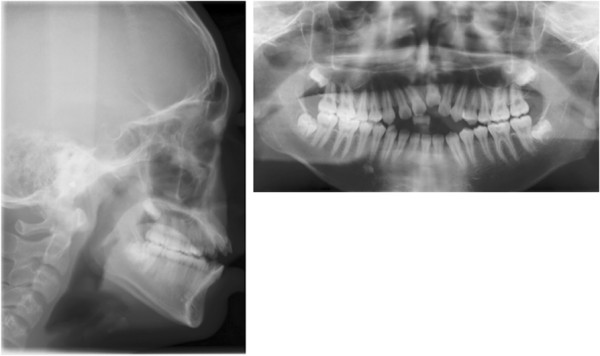
**Panoramic and cephalometric radiographs before treatment.**

A panoramic radiograph showed that the maxillary left central incisor was infra-occluded and the alveolar process in this region was deficient in vertical development. Cephalometric analysis showed a normal skeletal relationship with an ANB angle of 3.7° and a high mandibular plane angle of 39.11°. The maxillary and mandibular incisors were protruded: U1 to PP, 127.90°; and IMPA, 99.62°. The overbite was -8.87 mm (Figure 3, Table 1).

**Table 1:** **Cephalometric summary**

Measurement	Chinese population standards	Pretreatment	Posttreatment
SNA	82.8 ± 4.00	79.11	80.94
SNB	80.10 ± 3.90	75.41	76.49
ANB	2.70 ± 2.00	3.70	4.45
Overbite	2.50 ± 2.00	-8.87	1.75
MP/FH	31.10 ± 5.6	39.11	39.59
U1/PP	115.80 ± 5.70	127.90	110.22
IMPA	93.90 ± 6.20	99.62	78.08
ODI	72.80 ± 5.20	71.23	70.79
U1-PP	30.50 ± 2.10	29.78	32.03
U6-PP	26.20 ± 2.00	29.77	28.61
L1-MP	45.00 ± 2.10	42.21	48.09
L6-MP	35.80 ± 2.60	34.63	33.97

### Diagnosis, treatment objective, and treatment alternatives

This case was diagnosed as a skeletal Class I malocclusion with severe open bite and high mandibular plane angle. The maxillary left central incisor was diagnosed as potentially ankylosed because of the trauma history, infra-occlusion, inadequate alveolar bone in the maxillary anterior region, typical metallic sounds upon percussion, and lack of tooth mobility.

The treatment objectives were to: (1) correct the severe anterior open bite; (2) correct the labial inclination of the maxillary and mandibular incisors and reposition the intruded tooth; and (3) restore the alveolar bone defect.

Four treatment options were presented to the patient: (1) orthodontic treatment combined with luxation; (2) prosthetic buildup; (3) prosthetic buildup followed by orthodontic treatment; and (4) orthodontic treatment combined with segmental osteotomy. Risks and benefits of each procedure were explained in detail to the patient and his parents. The patient chose orthodontic treatment combined with luxation because he did not want to undergo surgery. The patient also agreed that option 4 would be considered if the first option failed.

### Treatment progress

**Figure 4:**
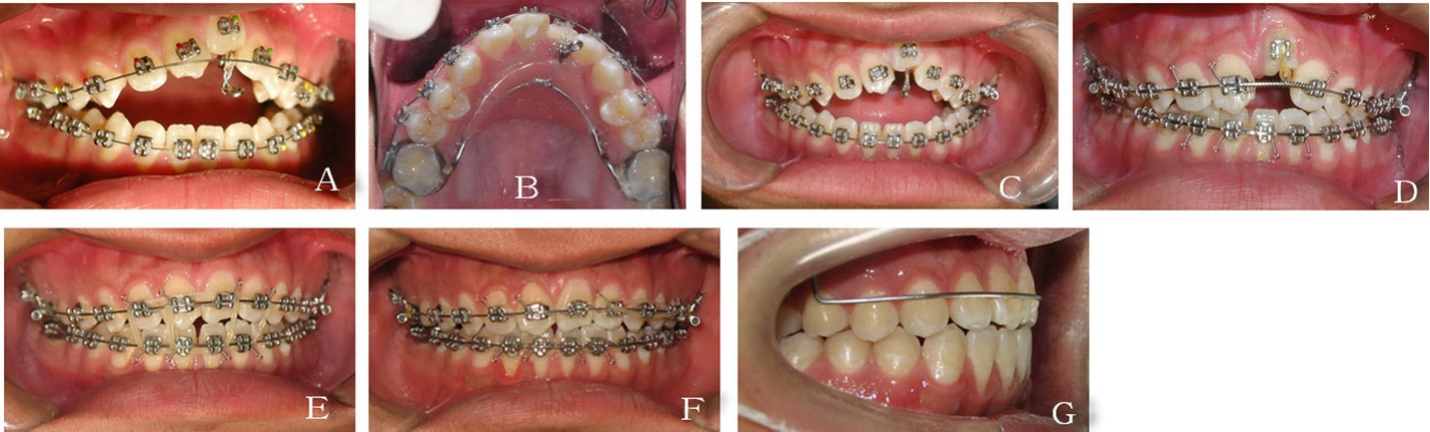
**Stages in the orthodontic tooth alignment. (A–F)** Orthodontic traction and alignment of the tooth; **(G)** A modified Hawley retainer bonded with resin to the central incisors to provide retention for the tooth in the dental arch.

**Figure 5:**
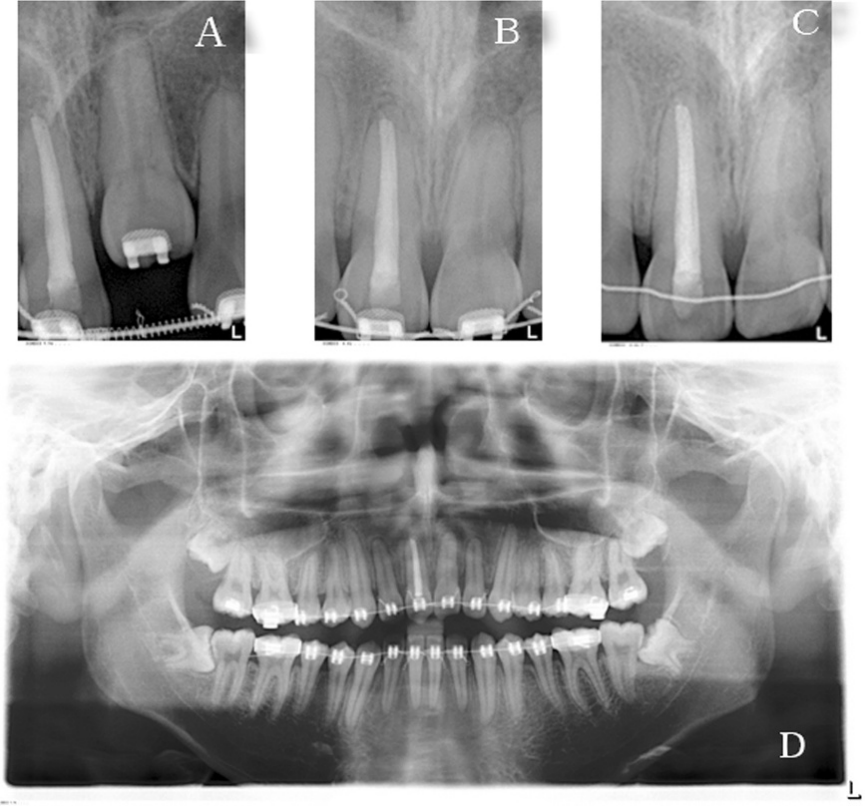
**Radiographic images of the tooth at the various treatment stages. (A)** Periapical radiograph showing the condition of the left central incisor was suitable for subluxation; **(B–C)** No resorption evident in the roots of the central incisors; **(D)** Panoramic radiograph taken to check the condition of the anterior teeth.

After the endodontist treated the right central incisor, maxillary and mandibular straight wire appliances (0.022 × 0.028 inch preadjusted) were placed (Figure 4A). A modified Nance appliance (Figure 4A and B) was used to extrude the left central incisor; however, no extrusion of the incisor had occurred after 8 months (Figure 4C). The diagnosis of ankylosis was confirmed, although the patient was absent from the clinic for 5 months during this period for personal reasons. Tooth alignment and leveling began with a 0.012 inch nickel-titanium archwire, followed by 0.014, 0.016 and 0.018 inch nickel titanium archwires. Final alignment was completed with a 0.019× 0.025 inch nickel-titanium archwire, followed by a 0.019 × 0.025 inch stainless steel archwire. In the twelfth month, upper accentuated-curve and lower reverse-curve rectangular stainless steel wires (0.019 × 0.025 inch) were placed after all teeth except the left central incisor were aligned. A push spring (0.012 inch, GRIKIN Advanced Materials Co. Ltd, China) was used to increase the space between the right central incisor and the left lateral incisor, then incisor tooth forceps were used to subluxate the left central incisor with torsional force. Orthodontic traction of the left central incisor was then applied with a power chain from the rectangular stainless steel arch wires. Vertical elastics were applied to decrease the open bite. In the nineteenth month, the open bite decreased markedly, but the infra-occlusion of the left central incisor worsened (Figure 4D). A second subluxation was suggested because the maxillary left central incisor appeared to have become re-ankylosed. Pulp vitality testing and periapical radiography (Figure 5A) showed that the condition of the left central incisor was adequate to withstand subluxation. Following the subluxation, orthodontic traction and application of vertical elastics was continued. Fourteen months later, the maxillary left central incisor had extruded to the correct position and the open bite was almost corrected (Figure 4E). A panoramic radiograph taken to check the condition of the anterior teeth found little evidence of root resorption (Figure 5D). A 0.016 inch upper accentuated-curve and lower reverse-curve made of Australian wire was used to treat the remaining open bite, which had been difficult to resolve using 0.019 × 0.025 inch rectangular stainless steel wires. This procedure took 4 months (Figure 4F). Given the high incidence of relapse of open bite, vertical elastics were continued for 12 hours per day until 6 months after the open bite had been corrected, and fixed retainers were chosen for both arches. During the treatment, the patient undertook orofacial myofunctional therapy. A modified Hawley retainer bonded with resin to the labial surface of the central incisors was used to prevent intrusion of the maxillary anterior teeth (Figure 4G).

### Treatment results

**Figure 6:**
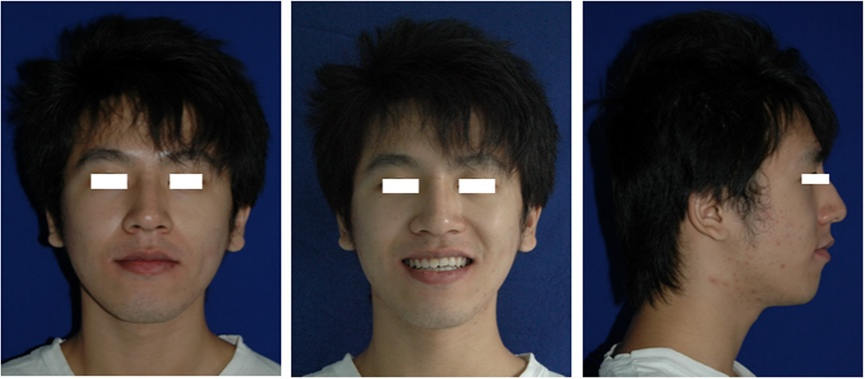
**Post-treatment facial photographs.**

**Figure 7:**
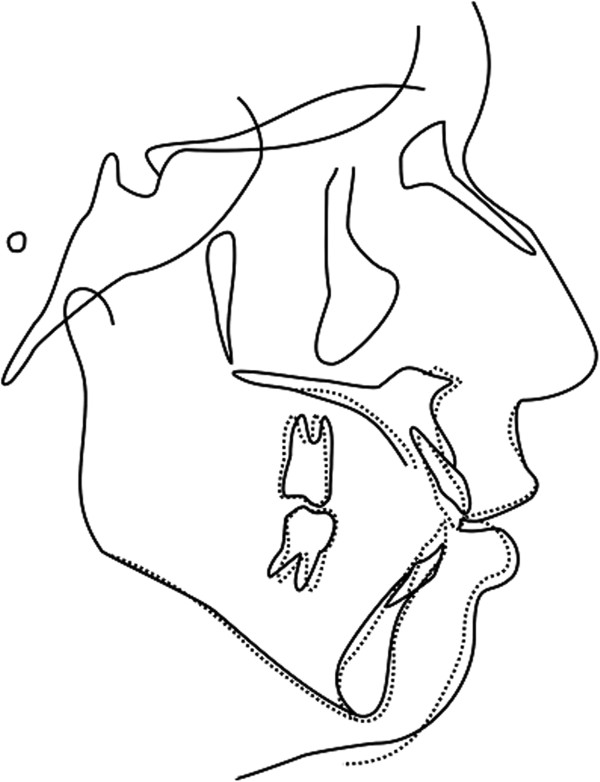
**Cephalometric superimposition registered on the sella-nasion line.** Solid: Pre-treatment; Dotted: Post-treatment.

**Figure 8:**
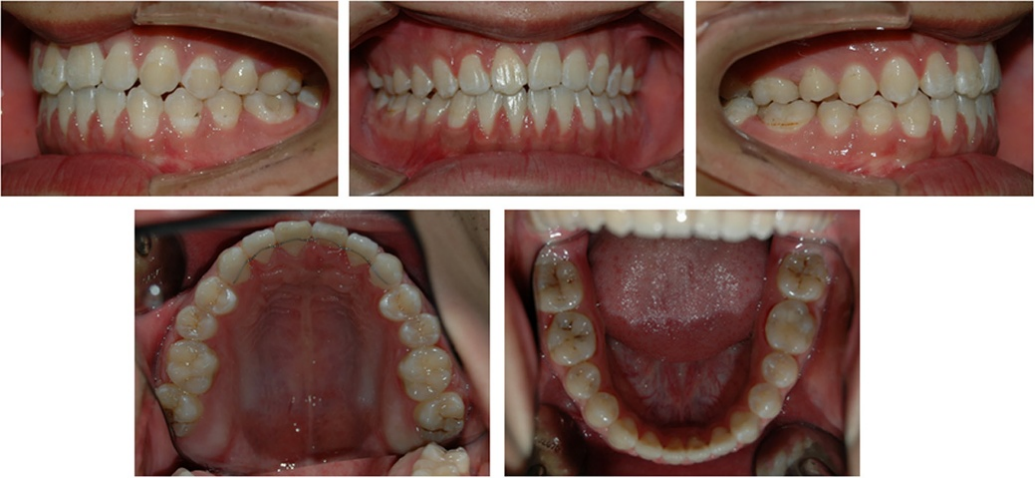
**Post-treatment intraoral photographs.**

**Figure 9:**
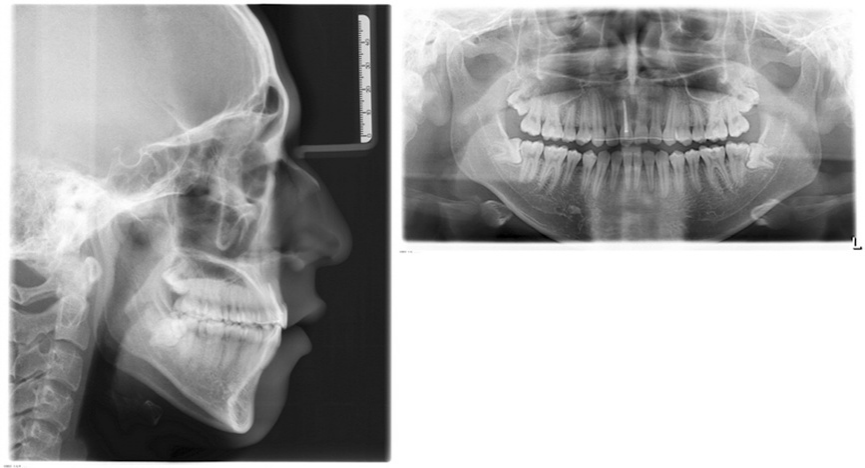
**Post-treatment radiographs.**

**Figure 10:**
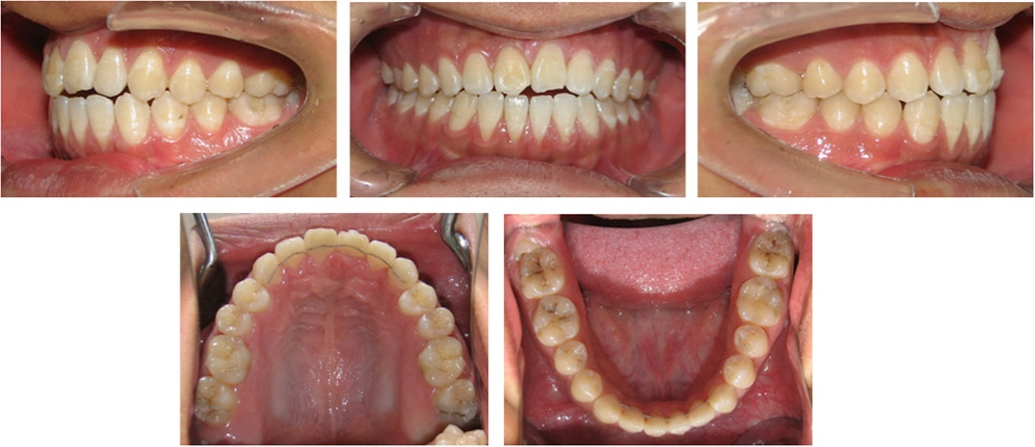
**Intraoral photographs of patient at 2-year follow-up.**

Post-treatment records (Figure 6) showed an improved profile resulting from the change in inclination of the maxillary and mandibular incisors. The maxillary central incisor to palatal plane decreased (127.9° to 110.22°), as did the mandibular central incisor to mandibular plane (99.62° to 78.08°). The overbite changed from -8.87 to 1.75 mm. The first molars were intruded and the maxillary central incisors were extruded (Table 1, Figure 7) .The molars were in a Class I relationship with normal overjet and overbite (Figure 8). The severe anterior open bite was corrected, as was the habit of tongue thrusting. Good root parallelism was observed on the post-treatment panoramic radiograph, with little evidence of incisal root resorption (Figure 9A). The pulp vitality of the left central incisor was retained. Two years post-retention (Figure 10), the occlusion was still in a Class I relationship. The periapical radiograph showed no root resorption in the central incisors (Figure 5C), and pulp vitality was retained. However, the overbite had decreased slightly.

## Discussion

It is difficult to predict whether dental ankylosis will occur after an accident, and it may not be noticed for several years in some cases. In a growing child, ankylosis can cause deleterious effects on occlusal development. Early diagnosis and an effective treatment plan are fundamental to preventing further eruption deviations and more severe malocclusion [5]. By missing out on early treatment, this patient developed progressive infra-occlusion of the ankylosed tooth and a defect in the vertical alveolar bone. The trauma occurred when the patient was 8 years old, at which time the damaged tooth probably had an open apex. Upon presentation eight years later, the tooth was in infra-occlusion but with no evidence of replacement resorption. This indicated that the periodontal ligament was vital. The pulp had revascularized with possible bone ingrowth and the root was locked by this bone into the alveolar bone.

Diagnosis of ankylosis on dental radiographs is often difficult, because the areas of ankylosis are small and may be invisible on the 2-dimensional image. The clinical diagnosis of ankylosis can be confirmed only when the affected tooth proves to be impossible to move [10, 20]. The patient in the present case had a history of trauma and infra-occlusion of the maxillary left central incisor. Therefore, the maxillary left central incisor was diagnosed as a potentially ankylosed tooth. To confirm the diagnosis, a modified Nance arch was used to pull the maxillary left central incisor. The benefit of this solution was that the reactive force did not affect the adjacent anterior teeth, but protected the anchorage, and it was easy to adjust the hook to pull the teeth in a normal direction.

Because of the vertical alveolar bone defect and the severe anterior open bite, it was thought that an esthetically acceptable result could not be achieved by treating the patient either with prosthetic buildup, extraction of the ankylosed tooth and restoration of the space with prosthetics or implants, or prosthetic buildup followed by orthodontic treatment. Orthodontic treatment combined with luxation was a possible approach in this case, in spite of risk factors including fracture, recurrence of the ankylosis, and the need for endodontic treatment [17]. Another alternative was orthodontic treatment combined with corticotomy and distraction osteogenesis [18, 19]. This would involve gradual distraction of the bony block along with the attached soft tissue to produce tissue regeneration; however, this patient was not willing to undergo surgery.

Temporary anchorage devices (mini-implants) are usually used to intrude molars to correct a severe anterior open bite; this solution can also minimize the relapse of open bite. This patient was uncomfortable with the idea of implants and rejected this option, although we explained the benefits of implants and the disadvantages of intermaxillary vertical elastics. We could treat the anterior alveolar bone defect with extrusive mechanics and improve the patient’s smile (so that he showed more incisors) by using intermaxillary vertical elastics to extrude the anterior incisors, so this was the option we chose, given that the patient had rejected surgery and his facial profile was good. Enacar et al. [21] used 0.016 × 0.022 inch upper accentuated-curve and lower reverse-curve nickel titanium arch wires with vertical elastics applied in the canine region to treat patients with an open bite. They suggested that the results were similar to those obtained by the multiloop edgewise arch wire system. We used 0.019 × 0.025 inch upper accentuated-curve and lower reverse-curve rectangular stainless steel wires to treat the open bite and simultaneously pull the ankylosed incisor which had been surgically luxated. We chose 0.019 × 0.025 inch rectangular stainless steel wires to avoid intrusion of the adjacent anchor teeth when the application of orthodontic forces failed to extrude the ankylosed tooth. During the treatment, the ankylosis recurred and a second luxation was performed. It was obvious that the damaged central incisor was stuck in the alveolus due to bone ingrowth. In such instances it is possible to break the bone in the apical area and afterwards extrude the tooth. The broken part of the bone in the apical area later heals with the surrounding bone and the tooth is stuck again. Surgical luxation of an ankylosed permanent tooth is recommended if no change is apparent after 6 months. Moreover, it is suggested that the tooth should be extracted if the second luxation is unsuccessful [22]. We asked the patient to undergo orofacial myofunctional therapy to assist in retention following treatment of the open bite [23]. The relapse of the overbite during retention may have been due to extrusion of the posterior teeth and intrusion of the anterior teeth, because the decrease in the open bite was attributed to intrusion of the posterior teeth and extrusion of the anterior teeth during treatment (Table 1). Potential bone growth could be another reason for the relapse. Relapse after anterior open bite treatment has been attributed to tongue posture, growth patterns, treatment parameters, and surgical fragment instability, possibly due to the increased facial height and extrusion of maxillary molars [24]. More than 35% of treated open-bite patients demonstrate a post-retention open bite of 3 mm or more [25].The importance of retention is to enhance stability, especially by eliminating the cause of the open bite. Special methods are needed for retention of open bite [26, 27].

## Conclusion

This case report illustrates an acceptable treatment result for a patient with an open bite and an ankylosed tooth. The approach chosen was surgical luxation with orthodontic traction, which was shown to be an effective approach in cases where the ankylosed tooth has a single root and the pulp is vital. However, the outcome of orthodontic traction cannot be predicted at the clinical treatment stage, and long-term monitoring of occlusal stability and maintenance of periodontal health are crucial factors in the post-treatment stage.

### Consent

Written informed consent was obtained from the patient for publication of this case report and any accompanying images. A copy of the written consent is available for review by the Editor-in-Chief of this journal.
